# Comprehensive Metabolomic, Lipidomic and Microscopic Profiling of *Yarrowia lipolytica* during Lipid Accumulation Identifies Targets for Increased Lipogenesis

**DOI:** 10.1371/journal.pone.0123188

**Published:** 2015-04-23

**Authors:** Kyle R. Pomraning, Siwei Wei, Sue A. Karagiosis, Young-Mo Kim, Alice C. Dohnalkova, Bruce W. Arey, Erin L. Bredeweg, Galya Orr, Thomas O. Metz, Scott E. Baker

**Affiliations:** 1 Environmental Molecular Sciences Laboratory, Pacific Northwest National Laboratory, Richland, WA, United States of America; 2 Fundamental and Computer Sciences Directorate, Pacific Northwest National Laboratory, Richland, WA, United States of America; 3 Energy and Environment Directorate, Pacific Northwest National Laboratory, Richland, WA, United States of America; Ruhr-University Bochum, GERMANY

## Abstract

*Yarrowia lipolytica* is an oleaginous ascomycete yeast that accumulates large amounts of lipids and has potential as a biofuel producing organism. Despite a growing scientific literature focused on lipid production by *Y*. *lipolytica*, there remain significant knowledge gaps regarding the key biological processes involved. We applied a combination of metabolomic and lipidomic profiling approaches as well as microscopic techniques to identify and characterize the key pathways involved in *de novo* lipid accumulation from glucose in batch cultured, wild-type *Y*. *lipolytica*. We found that lipids accumulated rapidly and peaked at 48 hours during the five day experiment, concurrent with a shift in amino acid metabolism. We also report that exhaustion of extracellular sugars coincided with thickening of the cell wall, suggesting that genes involved in cell wall biogenesis may be a useful target for improving the efficiency of lipid producing yeast strains.

## Introduction

With the increasing emphasis on sustainable energy sources and global warming, lipid derived biofuels have been proposed as a promising substitute for fossil fuels. As an alternative, most lipid derived biofuels are currently produced from the conversion of vegetable oils. This is less promising due to high production costs and competition with food supplies. Microbial oil production is an attractive alternative to vegetable oil due to advantages that include low land consumption and high efficiency [1]. *Yarrowia lipolytica*, an ascomycetous yeast species, has a history of use as a genetically tractable biotechnology organism and is an attractive source for bioproducts. It has been used for production of organic acids [2,3,4], proteins [5,6,7] and is an excellent cell factory for lipid production because of native lipid accumulation [8], easy engineering [9] and robust growth on a variety of substrates [10,11].

Understanding the molecular mechanisms involved in lipogenesis in *Y*. *lipolytica* will guide genetic engineering to increase lipid accumulation. The complete sequence of its genome revealed the metabolic pathway for *de novo* lipid synthesis [9,12,13] while transcriptome analysis has provided useful information about the dynamic biological processes during the transition from biomass production to lipid accumulation [14], verifying that rerouting of carbon fluxes upon nitrogen limitation causes lipid accumulation. Studying lipids and small metabolites provides more direct information regarding relevant metabolic pathways and instant changes in concentrations within cells. Using metabolomics and lipidomics approaches to study *Y*. *lipolytica* could give insights into the metabolic processes involved in lipid production and be adapted to other oleaginous yeasts. Metabolome and lipidome studies in *Y*. *lipolytica* have been limited. Among those published, Zhao et al. investigated metabolome extraction approaches [15], Christen and Sauer applied ^13^C flux analysis and metabolomics to understand aerobic glucose metabolism of seven yeasts including *Y*. *lipolytica* [16], and Hein and Hayen performed lipidomic profiling on *Y*. *lipolytica* to determine the composition of its glycerophospholipids [17]. However, these studies focused on subsets of the metabolome and lipidome, and none focused on the process of lipid production and accumulation that is vital to engineering this organism for use in commercial biofuel production.

Here we applied a comprehensive approach to characterize the metabolic processes associated with lipid accumulation in *Y*. *lipolytica* by growing cells for five days and collecting biomass and spent media at 12 hour intervals. During this time course, cell morphology changes were characterized by electron and helium ion microscopy, while confocal microscopy was used to monitor cell wall and lipid body growth. Intact lipids were analyzed by ultra-performance liquid chromatography-mass spectrometry (UPLC-MS) [18] and fatty acid profiling was performed using gas chromatography-mass spectrometry (GC-MS). Metabolomics analyses of polar metabolites were also performed using GC-MS. This is the first comprehensive metabolomics/lipidomics study performed on *Y*. *lipolytica*. We found that our approach greatly expanded our understanding of metabolism in *Y*. *lipolytica* by characterizing changing pool sizes of an unprecedented number of small molecules in this yeast.

## Materials and Methods

### Chemicals

All chemicals and reagents were purchased from Sigma-Aldrich (St. Louis, MO) unless otherwise noted. HPLC-grade methanol, chloroform and GC-grade hexane were purchased from Fisher Scientific (Fair Lawn, NJ). Zirconium/silica beads (0.1 mm) were purchased from Biospec Products (Bartlesville, OK). The water used was of Milli-Q grade purified by a Millipore (Bedford, MA) Milli-Q UV Purification System.

### Yeast strains and cultivation

Wild-type *Y*. *lipolytica* strain ATCC 20460^TM^ (American Type Tissue Culture, Manassas, VA) was used for all experiments and was maintained on YPD plates (1% yeast extract, 1% peptone, 2% glucose, 2% agar) at 28°C. Frozen stocks were maintained at -80°C in 25% glycerol. For five day lipid production experiments, *Y*. *lipolytica* was pregrown from glycerol stocks for 24 h in 25 mL YNB-R broth (1.7 g/L yeast nitrogen base without amino acids, 1.5 g/L yeast extract, 50 g/L glucose) [9] in baffled 250 mL shake flasks with closures open to gas exchange at 28°C and 200 rpm. The cultures were then diluted into 50 mL of YNB-R broth in baffled 250 mL shake flasks to achieve an OD_600_ of 0.05, followed by growth at 28°C and 200 rpm for 120 h in triplicate. Three flasks were sacrificed every 12 h and samples collected in triplicate.

### Confocal microscopy

Cells were fixed in 4% paraformadehyde for 30 min and then washed 3 times for 5 min each with phosphate buffered saline (PBS, 8 g/L NaCl, 2.56 g/L Na_2_HPO_4_·7H2O, 0.2 g/L KCL, 0.2 g/L KH_2_PO_4_, pH 7.4). Yeast were harvested by centrifugation for 1 min at 211 x g. The cells were stained with LipidTox Red (Life technologies, Grand Island, NY) and Calcofluor White for 30 min. Cells were visualized in PBS without additional washing with a Zeiss LSM710 confocal laser-scanning microscope (Carl Zeiss MicroImaging GmbH, Munchen, Germany) and Plan-Apochromate 63x/1.40 Oil DIC M27 objective. Images were processed and lipid body volumes calculated using imageJ [19].

### Transmission electron microscopy

High-pressure freezing (HPF) and automatic freeze substitution (AFS), followed by plastic embedding, were used to produce thin sections of *Y*. *lipolytica*. Cells were fixed in 2.5% glutaraldehyde in their respective 12 hour interval time points, pelleted by brief centrifugation with a quick-spin minicentrifuge and then 3 uL of the suspension was transferred into an HPF flat specimen carrier and frozen with a Leica EM PACT high-pressure freezer (Leica Microsystems Inc., Bannockburn, IL) at a typical rate of 1,700°C/s. The pods with compacted frozen cells were transferred under liquid nitrogen to the precooled AFS (EM AFS; Leica), and a protocol for cell fixation, water substitution by acetone, and a gradual warm-up to RT was followed (see S1 Table). After 72 h, the samples were released from the pods by a gentle liquid flow induction in the surrounding acetone. The samples were washed three times in acetone, gradually infiltrated with an ascending series of Spurr’s low-viscosity embedding media (Electron Microscopy Sciences, Hatfield, PA) (25%, 50%, 75%, and three 100% washes for 120 min each), and cured at 60°C for 24 h. The polymerized blocks were sectioned to 70-nm thin sections with a Leica Ultracut UCT ultramicrotome, mounted on Formvar-coated 100 mesh Cu TEM grids sputtered with carbon, and poststained for 7 min with aqueous 2% uranyl acetate followed by 3 min of Reynolds’ lead citrate prior to TEM imaging. Samples were examined with the Tecnai T-12 transmission electron microscope (FEI) operating at 120 kV with an LaB6 filament. Images were collected digitally with a 2x2K Ultrascan 1000 CCD. DigitalMicrograph (Gatan) software was used for imaging and analyses of cellular features.

### Helium ion microscopy

Cell pellets were fixed in 2.5% glutaraldehyde overnight before being washed 3 times in PBS and serially dehydrated in ethanol. Cells were then critical point dried and placed onto an aluminum stub with carbon tape. The cells where carefully placed onto the carbon tape and then placed into a carbon sputter coater, ~5nm of carbon was sputtered onto the cells for conductivity. The Helium Ion Microscope conditions used where 35keV with 0.5pA current with a working distance ~8mm. All images were collected using a line scan averaging to minimize ion dose to the cells.

### Sample preparation for metabolome and lipidome analysis

For intracellular, polar metabolites, 1 mL of culture was added to 5 mL 60% (v/v) methanol at -20°C. The cells were then centrifuged (8000 × g, 4°C, 5 min). The supernatant was removed, and the cells were flash frozen in liquid nitrogen. The cells were then dried in a vacuum concentrator (Savant SpeedVac concentrator, Thermo Scientific) and 100 μL of nanopure water was added to dry cells prior to vortexing. Metabolites were extracted with 400 μL of chloroform/methanol mixture (2:1, v/v). After centrifugation (15,000 × g, 4°C, 5 min) the aqueous layer was transferred to glass vials and dried in a vacuum concentrator.

For extracellular metabolites, 1 mL of supernatant was collected after centrifugation of the culture (22,000 × g, 4°C, 5 min) and frozen in liquid nitrogen. From this, 20 μL spent medium was dried *in vacuo* and kept in -80°C before chemical derivatization.

For lipid analysis, 10 mL of culture was collected in a pre-weighed conical tube. The cells were centrifuged (8,000 × g, 20°C, 5 min) and the supernatant removed prior to lyophilization and determination of dry weight. About 10 mg of dry yeast was weighed prior to resuspension with 100 μL of nanopure water, to which was added a half volume of 0.1 mm zirconium/silica beads. Bead beating was performed for 5 min, and metabolites were extracted with 400 μL of chloroform/methanol mixture (2:1, v/v). After centrifugation (15,000 × g, 4°C, 5 min), the chloroform layer was transferred to glass vials and dried *in vacuo*.

### Chemical derivatization and GC-MS analysis

Polar metabolites were derivatized as described previously [20]. In short, 20 μL of methoxyamine in pyridine (30 mg/mL) were added to each sample, followed by incubation at 37°C with shaking for 90 min. Next, 80 μL of N-methyl-N-(trimethylsilyl)trifluoroacetamide (MSTFA) with 1% trimethylchlorosilane (TMCS) were added to each vial, followed by incubation at 37°C with shaking for 30 min. The samples were allowed to cool to room temperature and were then analyzed by GC-MS in random order.

Half of each lipid extract was removed and combined with 500 μL of 1.25 M HCl in methanol and kept at 80°C for 3 h to release free fatty acids and convert them into fatty acid methyl esters (FAMEs). Next, 500 μL of hexane was added to extract FAMEs, followed by addition of 500 μL water to the methanol layer to facilitate extraction. After centrifuging for 5 min, the hexane layer was collected for GC-MS analysis, and the samples were analyzed in random order.

An Agilent GC 7890A coupled with a single quadrupole MSD 5975C (Agilent Technologies, Inc, Santa Clara, CA) system was used for all analyses, and separations were performed using a HP-5MS column (30 m × 0.25 mm × 0.25 μm; Agilent Technologies, Inc.). The sample injection mode was splitless, and the injection volume was 1 μL. The GC oven was held at 60°C for 1 min after injection, and then increased to 325°C by 10°C/min, followed by a 5 min hold at 325°C. The injection port temperature was held at 250°C throughout the analysis.

### GC-MS data-processing

For FAMEs data, peaks were manually identified by comparing with a mixture of FAMEs standards (C8–C28) and the areas integrated. Normalization of peak areas by sample dry weight was then performed.

GC-MS raw data files were processed using MetaboliteDetector [21] for the analysis of intracellular and extracellular polar metabolites. In short, retention indices (RI) of detected metabolites were calculated based on analyses of a mixture of FAMEs standards (C8–C28), followed by their chromatographic alignment across all analyses after deconvolution. Metabolites were then identified by matching GC-MS features (characterized by measured retention indices and mass spectra) to the Agilent Fiehn Metabolomics Retention Time Locked (RTL) Library [22]. All metabolite identifications were manually validated to reduce deconvolution errors during automated data processing and to eliminate false identifications.

Subsequent data analyses were performed using the matrix of identified metabolites and unidentified features (characterized by retention indices and mass spectra) and their associated peak areas for intracellular and extracellular polar metabolites, and using the matrices of identified FAMEs and lipids and their associated peak areas. These matrices were z-score transformed and loaded into DAnTE [23] for visualization and multivariate analyses. Principal component analyses (PCA), hierarchical cluster analysis and Euclidian cluster analysis were performed to assess the reproducibility of the experiment and to identify natural clustering within the data. ANOVA with Tukey’s HSD test were used to assess significance of changes.

### LC-MS-based lipidomics analysis and data-processing

The remaining halves of the lipid extracts were reconstituted in 100 μL of isopropanol and were analyzed using a combined top-down/bottom-up UPLC-MS/MS-based lipidomics approach [18]. Briefly, a capillary column was slurry packed with HSS T3 particles (5 μm; Waters Corporation, Milford, MA), connected to a Waters NanoAcquity UPLC system and maintained at 40°C. One μL of sample was loaded onto a small trapping column (180 μm i.d.×2 cm) packed with reversed-phase particles (Symmetry C18, 5 μm; Waters) under the following isocratic conditions: 93% acetonitrile/water (40:60, v/v) containing 10 mM ammonium acetate (solvent A) and 7% acetonitrile/isopropanol (10:90, v/v) containing 10 mM ammonium acetate (solvent B). The lipids retained on the trapping column were then forward-flushed to the analytical column using the gradient as follows: t = 0 min, 10% B; t = 2 min,30% B; t = 10 min, 40% B; t = 20 min, 55% B; t = 40 min, 60% B; t = 70 min, 99.5% B; t = 90 min, 99.5% B; t = 95 min, 60% B; t = 97 min, 60% B; t = 98 min, 99.5% B; t = 100 min, 99.5% B; t = 102 min, 10% B; t = 130 min, 10% B. The flow rate was a constant 1.0 μL/min.

The LC system was interfaced to an LTQ-Velos Orbitrap mass spectrometer (Thermo Scientific, San Jose, CA) outfitted with a custom electrospray ionization (ESI) interface. Electrospray emitters were custom made by chemically etching 150 μm o.d. x 20 μm i.d. fused silica [24]. The heated capillary temperature and spray voltage were 350°C and 2.2 kV, respectively. Data-dependent MS/MS scan events were performed in the ion-trap (collision-induced dissociation; CID) or Orbitrap (HCD) using normalized collision energies (NCE) of 35 and 30 arbitrary units, respectively. Both CID and HCD were set with a maximum charge state of 2 and an isolation width of 2 *m/z* units. An activation Q value of 0.18 was used for CID. The full scan mass ranges for positive and negative ESI modes were 200–2,000 *m/z*, respectively. A dynamic exclusion time of 60 s was used to discriminate against previously analyzed ions using a -0.55 to 1.55 Da mass window. Each sample was analyzed in both positive and negative ESI modes.

Data were acquired under the control of Thermo Xcalibur software (Thermo Scientific) and high resolution MS scans in raw data files were subjected to deisotoping using Decon2LS [25]. LC-MS features were identified and then chromatographically aligned across all replicates for each sample using MultiAlign [26], and detected lipids were identified manually. All lipid peak areas were normalized by sample dry weight. Principal component analyses (PCA), hierarchical cluster analysis and Euclidian cluster analysis were performed to assess the reproducibility of the experiment and to identify natural clustering within the data. ANOVA with Tukey’s HSD test were used to assess significance of changes.

## Results

### Microscopy

**Fig 1:**
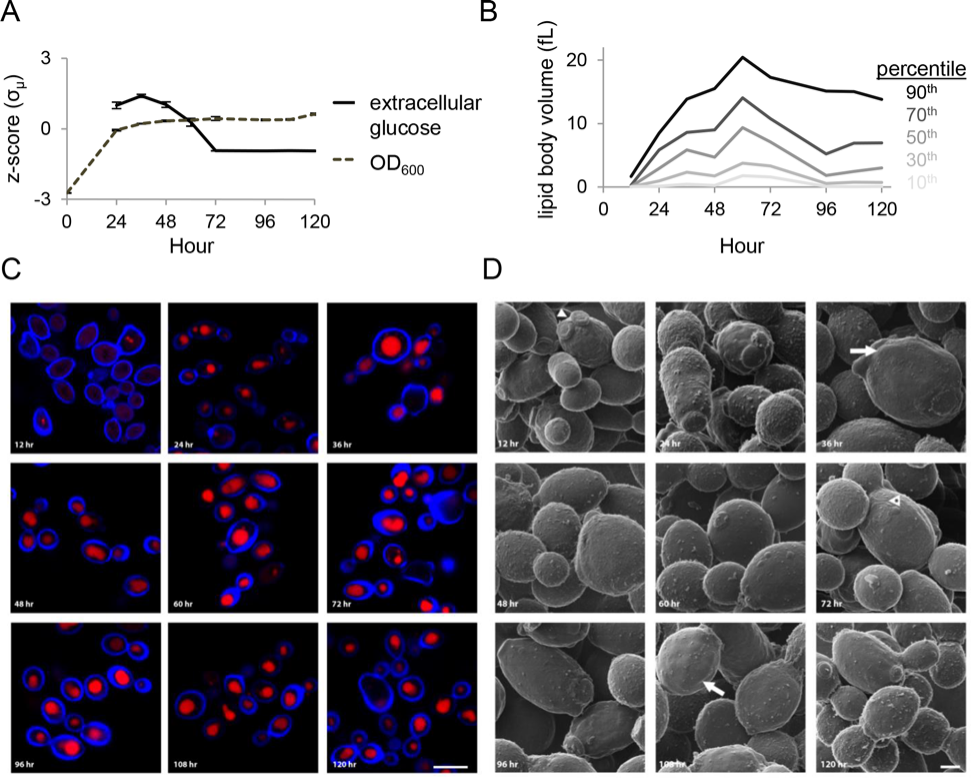
Lipid accumulation over the course of five days during *Y*. *lipolytica* batch culture. (A) Wild-type *Y*. *lipolytica* (ATCC 20460^TM^) was grown in YNB-R medium for five days with samples collected at 12 h intervals. Extracellular glucose was exhausted by 72 h. (B) The volume of lipid bodies was calculated from z-stack images and binned into percentiles to indicate the size distribution. (C) Scanning laser confocal microscopy of cells stained for neutral lipids (red) and cell wall (blue) reveal lipid bodies making up much of the intracellular space. Scale bar: 5 μm. (D) Helium ion microscopy reveals detailed cell surface structure. Arrowhead denotes typical bud scarring, arrowhead with asterisks highlights unusual bud scar morphology and arrows show areas of lumpy cell wall characteristic of cells of 36 h and older age. Scale bar: 1 μm.

**Fig 2:**
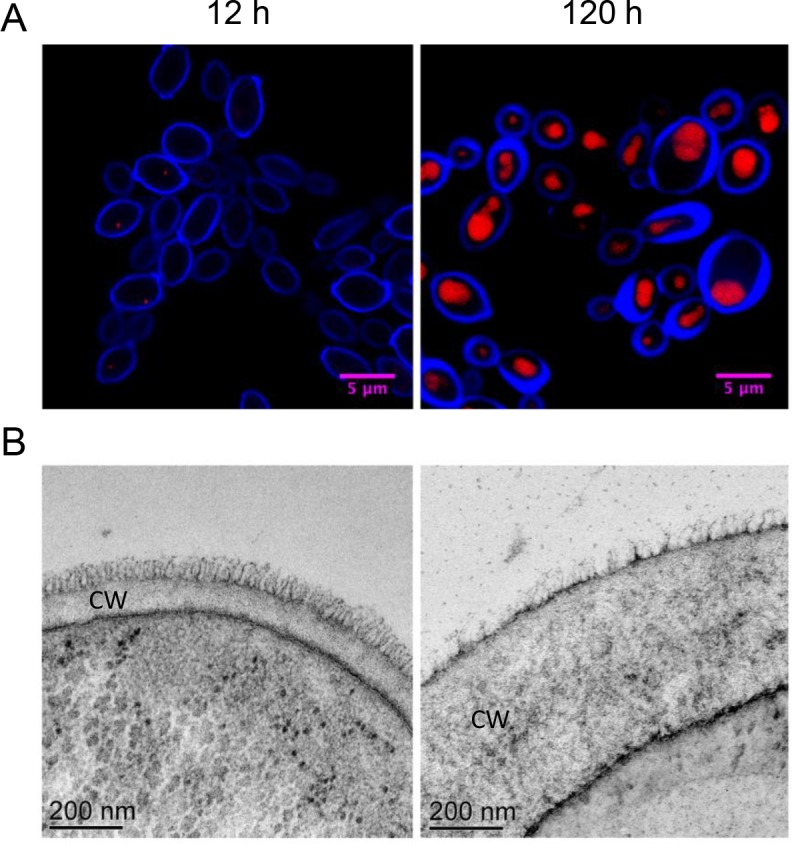
Transmission electron microscopy shows cell wall thickening during lipid accumulation. Both (A) confocal laser scanning microscopy and TEM show thickened cell wall morphology in later time points. Cells were stained for cell wall (blue) and lipid bodies (red) in (A). CW indicates the cell wall in (B).

We collected samples of *Y*. *lipolytica* grown in batch culture at 12 hour intervals for 120 hours. The cultures grew to near maximum cell density by 24 hours and depleted the main carbon source, glucose, from the medium by 72 hours (Fig 1A). We fixed cells and utilized scanning laser confocal microscopy to observe their morphological characteristics during lipid accumulation. Neutral lipid staining revealed that lipid bodies are small and make up very little of the intracellular space at 12 hours post-inoculation when the cells are presumably still growing exponentially (Fig 1 and S2 Table). Large lipid bodies are evident by 24 hours and by 60 hours nearly all the intracellular space stains for neutral lipids in most cells. We note that between 60 and 72 hours there is a significant change in the thickness of the cell wall, concurrent with depletion of glucose from the medium. To confirm this we analyzed cells using transmission electron microscopy and found that older cells have substantially thicker cell walls than young cells (Fig 2). We analyzed the cell surface and morphology using helium ion microscopy and found that the cells were generally plump and continuing to bud throughout the time course (Fig 1D). In contrast, cells stressed by nutrient limitation often appear elongated and grow as pseudo-filaments (data not shown).

### Intracellular and extracellular metabolite levels

**Fig 3:**
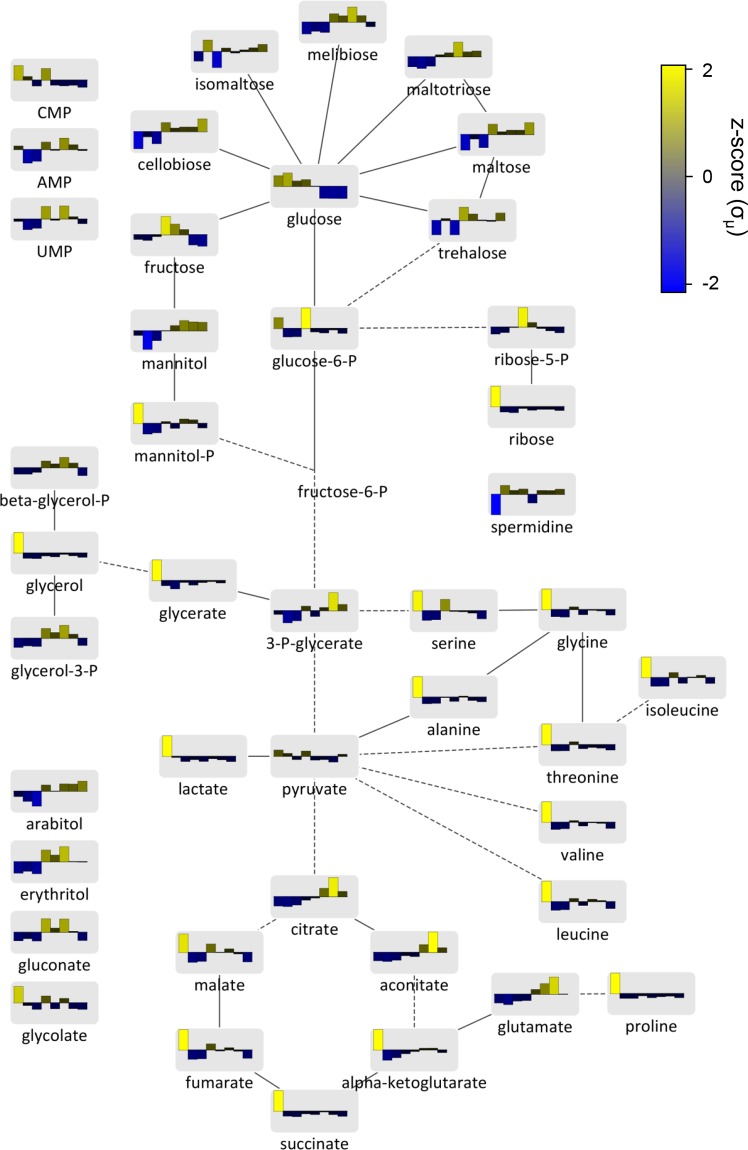
Intracellular metabolism is dynamic during lipid accumulation. A metabolic map including 43 measured intracellular metabolites was constructed based on the metabolic capabilities of the fungi *Saccharomyces cerevisiae* and *Neurospora crassa*. Each node represents a metabolite upon which median normalized z-scores for hours 24, 36, 48, 60, 72, 96, 108 and 120 are plotted left to right. Solid edges represent direct connections via an enzymatic reaction while dashed edges represent short sets of reactions where we did not measure any intermediates.

**Fig 4:**
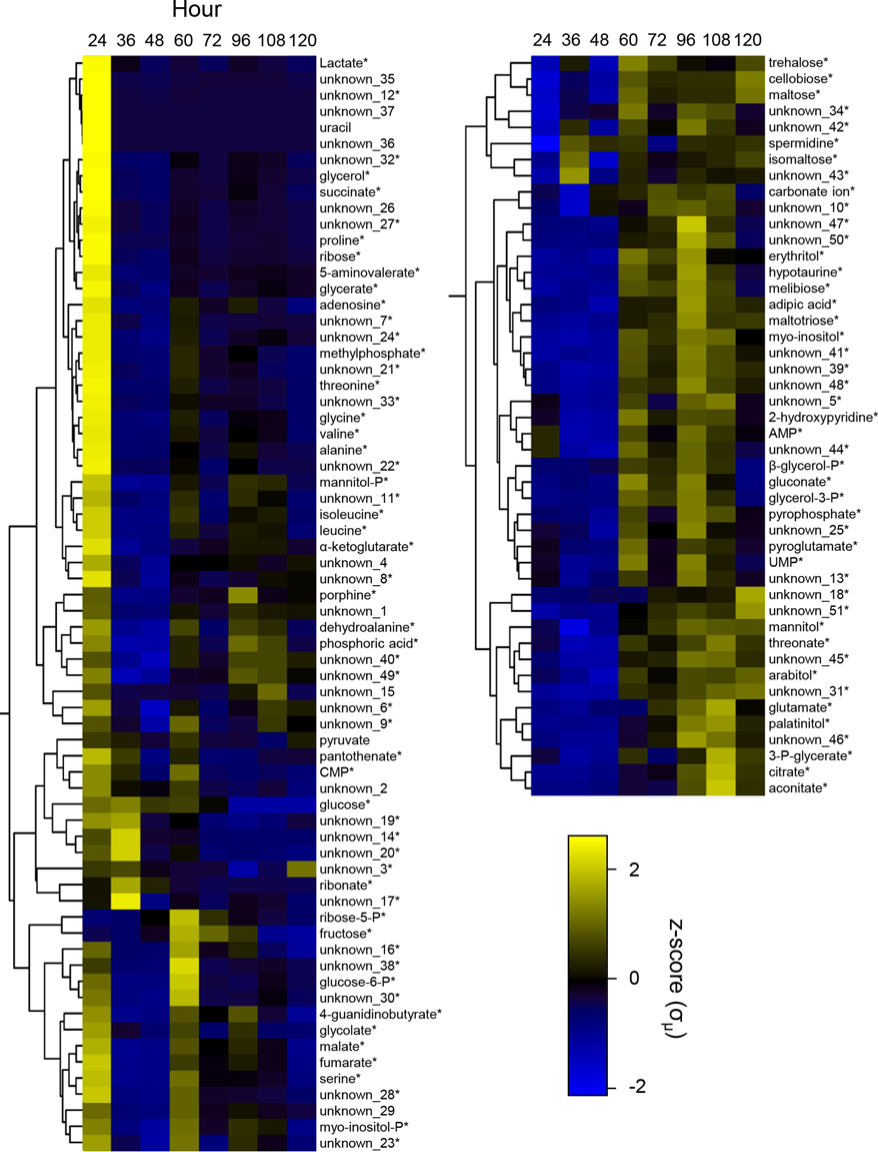
Clustering of metabolite profiles during lipid accumulation. Intracellular polar metabolite z-scores were clustered using a Euclidian approach to identify those that are metabolically linked. Z-scores are normalized by dry cell mass. *Metabolites that changed significantly during the time course by ANOVA (p < 0.01).

In order to monitor intracellular metabolic changes during the time course, we performed GC-MS-based metabolomics analyses, detecting 114 unique metabolite features, with 63 of these identified by matching to entries in the Agilent Fiehn Metabolomics RTL and NIST GC-MS libraries. Sixty one of 63 identified intracellular polar metabolites changed in concentration (p < 0.01), the exceptions being uracil and pyruvate. Intracellular, polar metabolite z-scores were overlaid on a metabolic map detailing aspects of central carbon metabolism in *Y*. *lipolytica* (Fig 3). Identified and unidentified intracellular metabolite z-score profiles were then clustered to group identified metabolites that behave similarly and unidentified metabolites that may be involved in related responses (Fig 4). Clustering analysis suggests that a variety of closely linked metabolites, for example, malate and fumarate or glycine and alanine, have similar abundance levels at each time point. Many of the metabolites (glycerol, glycerate, glycolate, lactate, ribose, mannitol-P, malate, fumarate, succinate α-ketoglutarate and all amino acids except glutamate) are detected at much higher levels at 24 h than any other time point (Figs 3 and 4). This may be due to their high starting concentration in the yeast extract containing medium or may be a signature of rapidly dividing cells.

A major shift in metabolism is seen during lipid accumulation between 24 and 48 h within the observed amino acid, glycolytic and TCA cycle metabolites. From 24 to 36 h, all observed amino acids (alanine, glycine, isoleucine, leucine, proline, serine, threonine, glutamate and valine), TCA cycle intermediates (α-ketoglutarate, fumarate, malate, succinate, citrate and aconitate) and glycolysis intermediates (glucose-6-P, 3-P-glycerate and pyruvate) decrease or do not change in relative concentration if they are already at their lowest concentration during the time course (Fig 3). This is concurrent with increasing intracellular levels of the glycolysis start point, glucose.

Interestingly, from 36 to 108 h, the intracellular level of glutamate gradually increases along with the closely linked TCA cycle intermediate α-ketoglutarate (Fig 3). Although we did not measure intracellular or extracellular ammonium levels during this experiment, we speculate that the increase in glutamate indicates the cells are beginning to fix the major nitrogen source ammonium sulfate by transamination of α-ketoglutarate to form glutamate at the expense of NADPH [27]. This is supported by minimal levels of free amino acids, a preferable nitrogen source present in the yeast extract containing medium, at hour 36; though, this may also indicate a high rate of protein synthesis. By hour 60, all free amino acid levels recover with the exception of glutamate suggesting it is being used as the nitrogen source for biosynthesis of serine, glycine, alanine, isoleucine, threonine, valine, proline and leucine.

**Fig 5:**
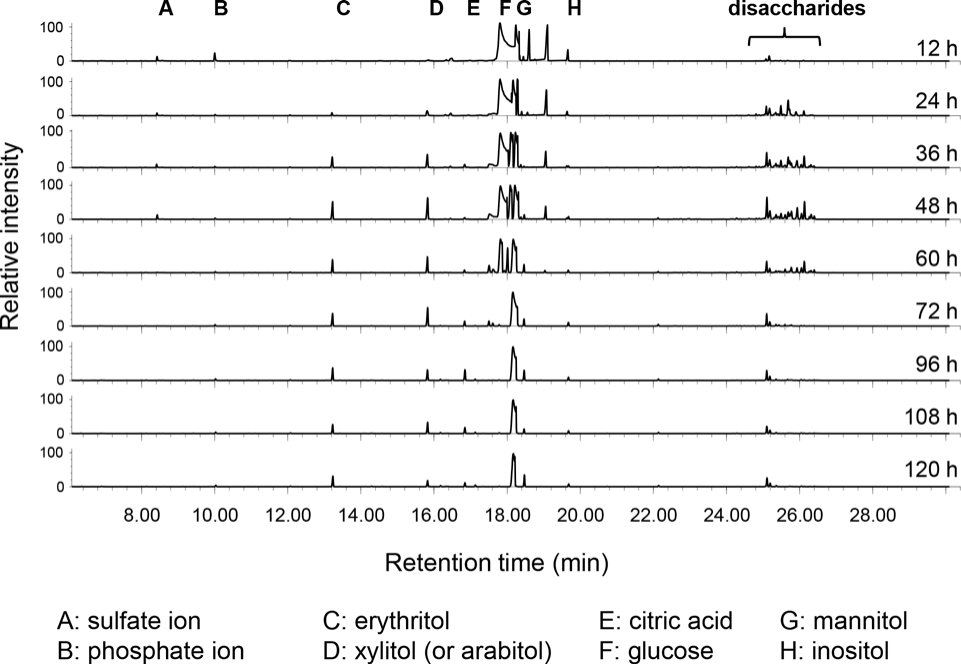
Extracellular metabolites during lipid accumulation. 89 metabolites were identified by GC-MS profiling in the medium after removal of the cells. Representative chromatographs are shown with peaks for identified metabolites indicated. Note the rapid appearance and disappearance of peaks representing a variety of disaccharides.

For extracellular metabolites, we detected 89 unique metabolite features, 18 of which were matched with the Agilent Fiehn library. Representative chromatograms of extracellular metabolite analysis across the time course are shown in Fig 5. Erythritol, a useful food additive that can be made from glycerol feed stocks in *Y*. *lipolytica* [28,29], gradually accumulated over the time course. Glucose, the major carbon source in the medium, was depleted after 60 h, indicating *Y*. *lipolytica* had to alter its carbon metabolism between 60 and 72 h. Toward the end of lag phase at 24 h we observed a variety of disaccharides (trehalose, maltose, isomaltose and other unidentified sugars) that were not present in the medium at 12 h (Fig 5). These sugars are all detectable intracellularly (Fig 3) and represent sugars either excreted by *Y*. *lipolytica* or the breakdown product of complex carbohydrates present in the yeast extract [30]. Between hours 48 and 72, concurrent with glucose exhaustion, these extracellular disaccharides rapidly disappear. Intracellular levels of cellobiose, meliobiose, maltose, trehalose and maltotriose increase between hours 48 and 60 when the extracellular disaccharides disappear (Fig 3) suggesting they are transported into the cell intact rather than being digested to sugar monomers.

### FAMEs and intact lipids

**Fig 6:**
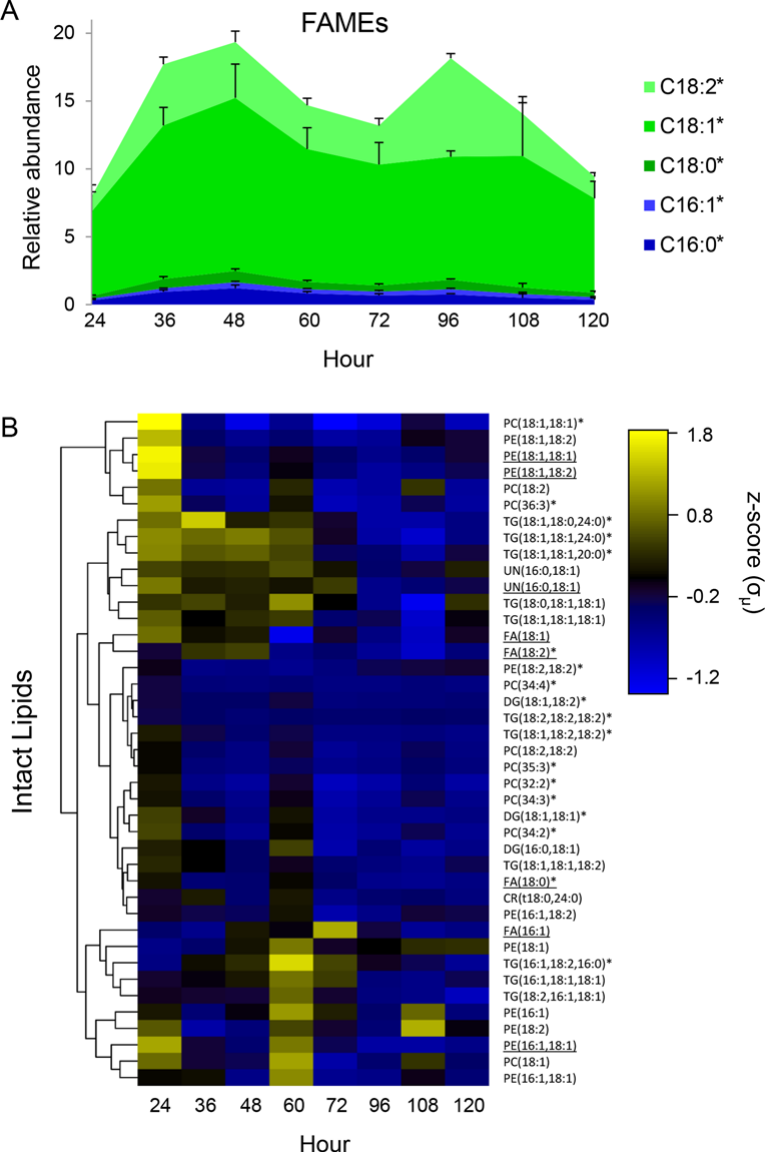
Lipid spectrum accumulated in batch culture is dynamic. Intracellular FAMEs and intact lipids were extracted at 12 hour intervals. (A) Extractable FAMEs accumulate to a maximum level at 48 h and drop in concentration as extracellular glucose is depleted. (B) Intact lipid z-scores were clustered using a Euclidian approach. PE, phosphatidylethanolamine; PC, phosphatidylcholine; TG, triacylglycerol; DG, diacylglycerol; CR, ceramide; FA, free fatty acid; UN, unknown headgroup. Underlines indicate intact lipids identified in negative ESI mode while lack of an underline indicates those identified in positive ESI mode. *Intact lipids that changed significantly during the time course by ANOVA (p < 0.01).

We analyzed FAMEs by GC-MS and primarily detected 16:1, 16:0, 18:2, 18:1, 18:0 and some minor 20:0, 22:0, 24:0 fatty acid chains in our samples (Fig 6A) similar to what has been described previously for wild-type *Y*. *lipolytica* [9,31,32]. All FAMEs change in concentration during the time course (p < 0.01). For intact lipids, we identified 33 lipids by positive ESI and 8 by negative ESI (Fig 6B), 18 of which change in concentration during the time course (p < 0.01). Lipid species detected by both modes of operation cluster similarly. The majority of the lipid species identified were from the diacylglycerol (DAG) and triacylglycerol (TAG) classes, which are the major lipid components of lipid bodies [33] and two glycerophospholipids, phosphatidylcholine (PC) and phosphatidylethanolamine (PE), which are major constituents of the phospholipid bilayer membranes. The fatty acid composition of the intact lipids is similar to that seen from the FAMEs. The FAMEs are dominated by mono- and di-unsaturated 18C species that increase to their maximum level at 48 h and decrease to a steady level around 72 h (Fig 6A). Smaller but significant contributions come from 16:0, 16:1 and 18:0 species. The distribution of FAMEs is not constant. At 24 h C18:1 FAMEs make up 78% (mole fraction) of the total but are diluted by rapidly increasing levels of C16:0, C16:1, C18:0 and C18:2 FAMEs to 66% (mole fraction) by hour 48 (Fig 6A).

## Discussion

The yeast *Y*. *lipolytica* is a popular species for industrial microbiology and for studying basic research questions related to its ability to accumulate lipids. Human dependence on fossil fuels is extending *Y*. *lipolytica*’s industrial uses, as it is being widely considered for production of long chain fatty acids as biodiesel precursors [13,31,34]. It accumulates more nonpolar lipids in the form of di- and triacylglycerols than most yeast when grown on glucose as a sole carbon source, which categorizes *Y*. *lipolytica* as an oleaginous yeast [32,35]. General features of metabolism that differ between oleaginous and non-oleaginous fungi have been reviewed previously [34,36,37,38]. However, comprehensive metabolite analysis of *Y*. *lipolytica* has not been performed. In this study, we grew a wild-type strain of *Y*. *lipolytica* with a genetic background common to many strains used for research in previously defined lipid accumulation conditions [9]. We analyzed the phenotype at the level of the lipidome and metabolome and investigated changes in the cell structure associated with lipid accumulation using transmission electron-, helium ion-, and scanning laser confocal microscopy.

### Lipid accumulation

During the time course, *Y*. *lipolytica* had completed exponential growth by hour 24 after which the OD_600_ remained relatively constant (Fig 1A). At 12 h most cells had no or few small lipid bodies, but by 24 h many cells with large lipid bodies were present (Fig 1B and 1C), in agreement with studies of *S*. *cerevisiae* that found lipid bodies increase in size during late log phase and reach maximum size at maximum cell density [39]. However, saturation of most cells with large lipid bodies filling most of the intracellular space did not occur until 36 h and later.

FAME abundance is in general accordance with microscopic observations. That is, even numbered fatty acid chains from C16 to C24 in length are low in concentration at 24 h and significantly increase to their maximum concentration at 48 h (p < 0.05) (Fig 6A). Intact lipid data indicate that, from 24 to 48 h, the contribution of side chains from the major membrane lipids (phosphatidylcholine, phosphatidylethanolamine and diacylglycerol) generally decreases. However, we did not identify any intact lipids that increased significantly from 24 to 48 h, limiting our ability to determine which contributed to the changes seen with the FAMEs.

Both the quantity of FAMEs and the volume of the lipid bodies increase to their maximum at 48 and 60 h, respectively (Figs 1 and 6). This occurs after maximum cell density is reached revealing that lipid metabolism is quite dynamic even in stationary phase cells. We have observed that cells growing in rich YPD medium (10 g/L yeast extract, 10 g/L bacto peptone, 20 g/L glucose) tend not to accumulate large lipid bodies, even after long periods in stationary phase (data not shown). Thus we expect that differences in metabolism when growing in minimal versus rich medium induce lipogenesis. From our experiments, the most likely candidate for this difference is free amino acids. The YNB-R medium used has a low concentration of free amino acids that are readily available for protein synthesis and some of which yeast will utilize as a preferable source of nitrogen in addition to the more abundant source, ammonium [40,41]. Amino acids appear to be taken up rapidly by the cells, evidenced by the high level of all measured amino acids except glutamate at 24 h (Fig 3). Intracellular amino acid levels then drop between 24 and 36 h to their lowest point in the time course, suggesting the cells have utilized all free amino acids from the medium but have not altered their metabolism to produce them *de novo*. To do so the cells must begin fixing nitrogen from ammonium sulfate by converting α-ketoglutarate to glutamate and then glutamate to glutamine [42]. Amino acid levels remain low until at least 48 h, during which intracellular glucose levels remain high and lipogenesis is occurring. Thus, accumulation of lipids to their maximum level appears to occur while the cells are transitioning to *de novo* biosynthesis of amino acids in the presence of excess glucose.

From 48 to 72 h, extracellular glucose is rapidly utilized during which time the quantity of FAMEs followed by the volume of the lipid bodies decreases (Figs 1, 5 and 6). Understanding the mechanism of carbon loss from the lipid bodies is important if *Y*. *lipolytica* is to be used as an industrial lipid factory. From our data, we note that a variety of TCA cycle intermediates, sugars important for cell wall biosynthesis, and other alcohols, acids and unknown compounds accumulate during this time (Fig 4). However, absolute molar concentrations of metabolites along with flux measurements will be essential to directly determine the flow of carbon during these metabolic transitions.

### Extracellular sugar utilization

We observed extracellular accumulation of a wide variety of disaccharides during the time course (Fig 5). At this point it is unclear whether the extracellular disaccharides were excreted from the cells or represents breakdown products of more complex extracellular sugars. Extracellular disaccharides increased and decreased in concentration during a narrow time frame, which may represent a single metabolic burst followed by uptake after the preferable carbon source glucose was exhausted, or constant production and uptake of disaccharides at a relatively constant rate from 24 to 72 h. The fact that the intracellular levels of disaccharides are dynamic during the first 72 h of the experiment with ubiquitously low levels at hour 24 and high levels at hour 72, while the extracellular levels of disaccharides remain static from hour 24 to 60 (Figs 3 and 5), supports the former hypothesis that they were produced primarily in a short burst between 12 and 24 h and internalized only after glucose was exhausted.

Scanning laser confocal microscopy of cells stained for chitin indicated that the thickness of the cell walls may be changing during the time course. We were particularly intrigued by the change in cell wall thickness observed between 60 and 72 h when the extracellular disaccharides were depleted and used transmission electron microscopy to more directly observe the cell wall. We confirmed that after 120 h, many of the cells had a significantly thicker cell wall than those of cells collected at 12 h (Fig 2), suggesting that excessive carbon allocation to cell wall polymers may be an overlooked target for engineering *Y*. *lipolytica* for lipid accumulation, or for that matter, optimizing any yeast for maximum yield of carbon based products.

### Biofuel properties of FAMEs

The bulk of the isolated FAMEs, which would presumably serve as the source for biodiesel, are C16:0, C16:1, C18:0, C18:1 and C18:2 chains, as previously found for wild-type *Y*. *lipolytica* [33]. At lower, but still detectable levels are saturated C20:0, C22:0 and C24:0 chains. Saturated C16:0 and C18:0 chains generally have good fuel stability, lower NOx emission, higher ignition quality but lower cold flow properties than conventional diesel making them poor for cold weather, while C18:2 chains may be too unstable for use as a fuel [43]. The monounsaturated C16:1 and 18:1 chains have a cetane number above the lower acceptable limits in the US and EU [44] and exhibit biofuel properties that balance stability, emissions, ignition quality and cold weather flow [43]. We found the highest concentration of C18:1 FAMEs after 48 h of aerobic growth in shake flasks, however, at 24 h, C18:1 chains make up a higher fraction of the total FAMEs. This indicates that the biofuel quality of the lipids was highest prior to peak lipid accumulation; the main difference in FAME data between 24 and 48 h being the increase in C18:2 FAMEs (Fig 6A).

### Conclusions

In summary, we have used a variety of techniques to characterize lipid accumulation in the oleaginous yeast *Y*. *lipolytica* metabolically and microscopically. In our growth conditions we observed complex changes in intra- and extracellular metabolite levels during batch culture and correlate these with microscopically observed cellular features. Substantial effort has been applied in the past few years to understanding the nature of and engineering oleaginous yeast [9,10,13,14,31], but to date only limited work has been done to characterize lipid accumulation metabolically. Here we provide a comprehensive dataset describing lipid accumulation in *Y*. *lipolytica* that will enable more detailed experiments to understand the oleaginous nature of *Yarrowia* and provide genetic targets for future metabolic engineering efforts.

## Supporting Information

S1 Fig **A.** Principal component analysis of FAME datasets. Biological replicates are color coded and plotted along principal components 1 and 2 to visualize clustering and reproducibility. **B.** Principal component analysis of intact lipid datasets. Biological replicates are color coded and plotted along principal components 1 and 2 to visualize clustering and reproducibility. **C.** Principal component analysis of extracellular metabolite datasets. Biological replicates are color coded and plotted along principal components 1 and 2 to visualize clustering and reproducibility. **D.** Principal component analysis of intracellular polar metabolite datasets. Biological replicates are color coded and plotted along principal components 1 and 2 to visualize clustering and reproducibility.
(PDF)

S1 Table AFS procedure for *Y*. *lipolytica* cell suspensions. Substitution medium at T1 and T2 was 1% glutaraldehyde and 0.1% tannic acid in anhydrous acetone. Three washes in 100% acetone, and 1% osmium tetraoxide in 100% acetone followed as substitution media in T2–T3.
(PDF)

S2 Table Volume of lipid droplets. Measurements were obtained from z-stacks of cells stained for neutral lipids as in Fig 1. All volumes are in femtoliters.
(PDF)
