# Supplementary material for: Comprehensive Metabolomic, Lipidomic and Microscopic Profiling of Yarrowia lipolytica during Lipid Accumulation Identifies Targets for Increased Lipogenesis
Source: PLoS One. 2015 Apr 23;10(4):e0123188. doi: 10.1371/journal.pone.0123188 (PMC4408067; doi:10.1371/journal.pone.0123188)
Supplement: S1 Fig — A. Principal component analysis of FAME datasets. Biological replicates are color coded and plotted along principal components 1 and 2 to visualize clustering and reproducibility. B. Principal component analysis of intact lipid datasets. Biological replicates are color coded and plotted along principal components 1 and 2 to visualize clustering and reproducibility. C. Principal component analysis of extracellular metabolite datasets. Biological replicates are color coded and plotted along principal components 1 and 2 to visualize clustering and reproducibility. D. Principal component analysis of intracellular polar metabolite datasets. Biological replicates are color coded and plotted along principal components 1 and 2 to visualize clustering and reproducibility. (PDF) [file pone.0123188.s001.pdf]

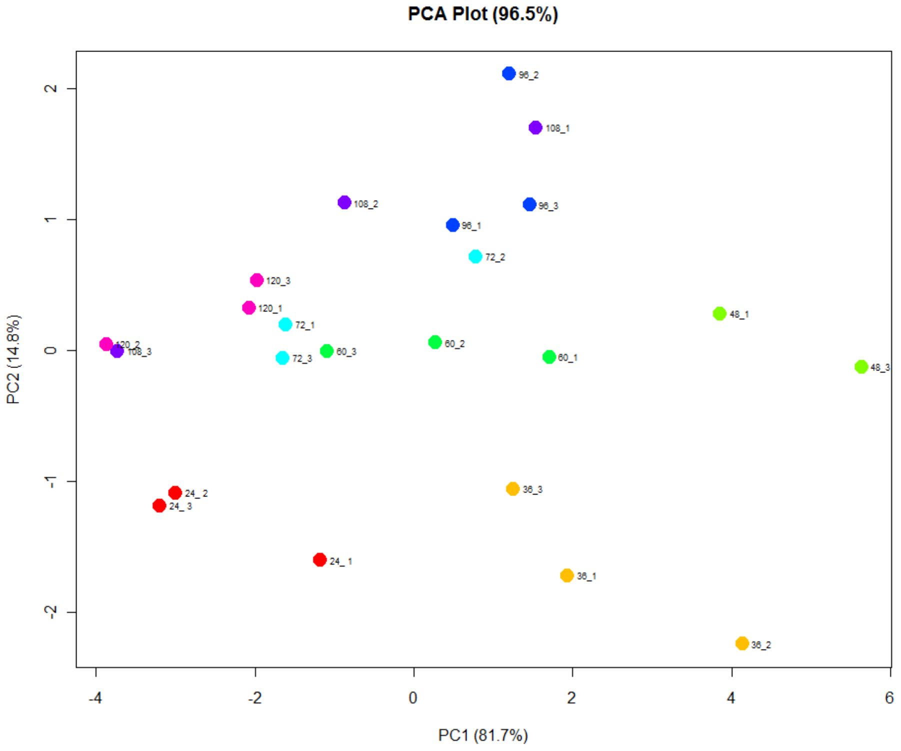

Supplemental figure 1A. Principal component analysis of FAME datasets. Biological replicates are color coded and plotted along principal components 1 and 2 to visualize clustering and reproducibility.

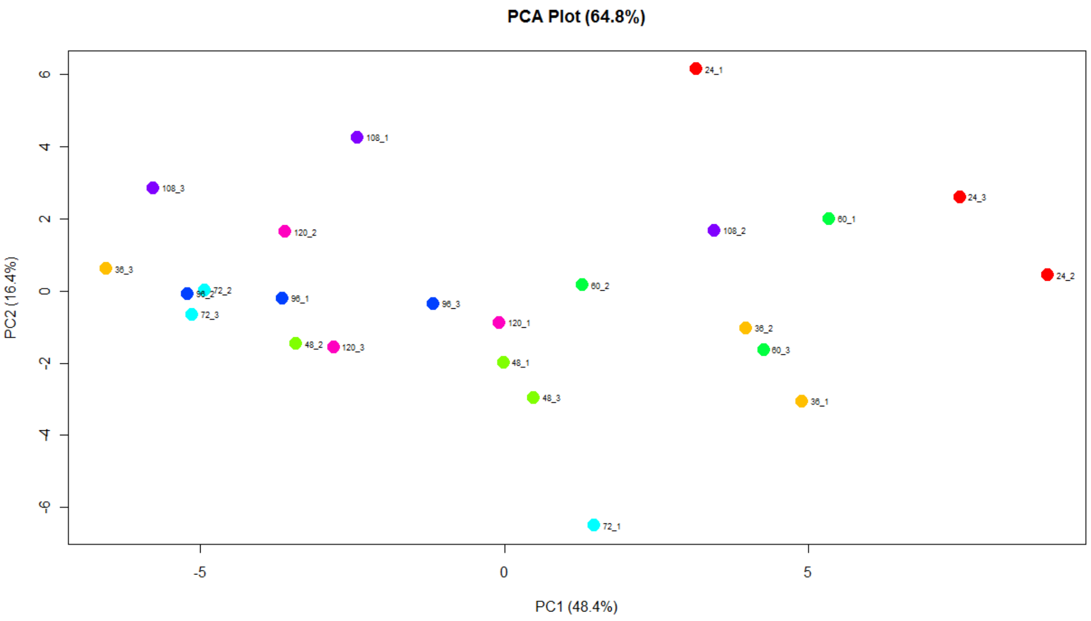

Supplemental figure 1B. Principal component analysis of intact lipid datasets. Biological replicates are color coded and plotted along principal components 1 and 2 to visualize clustering and reproducibility.

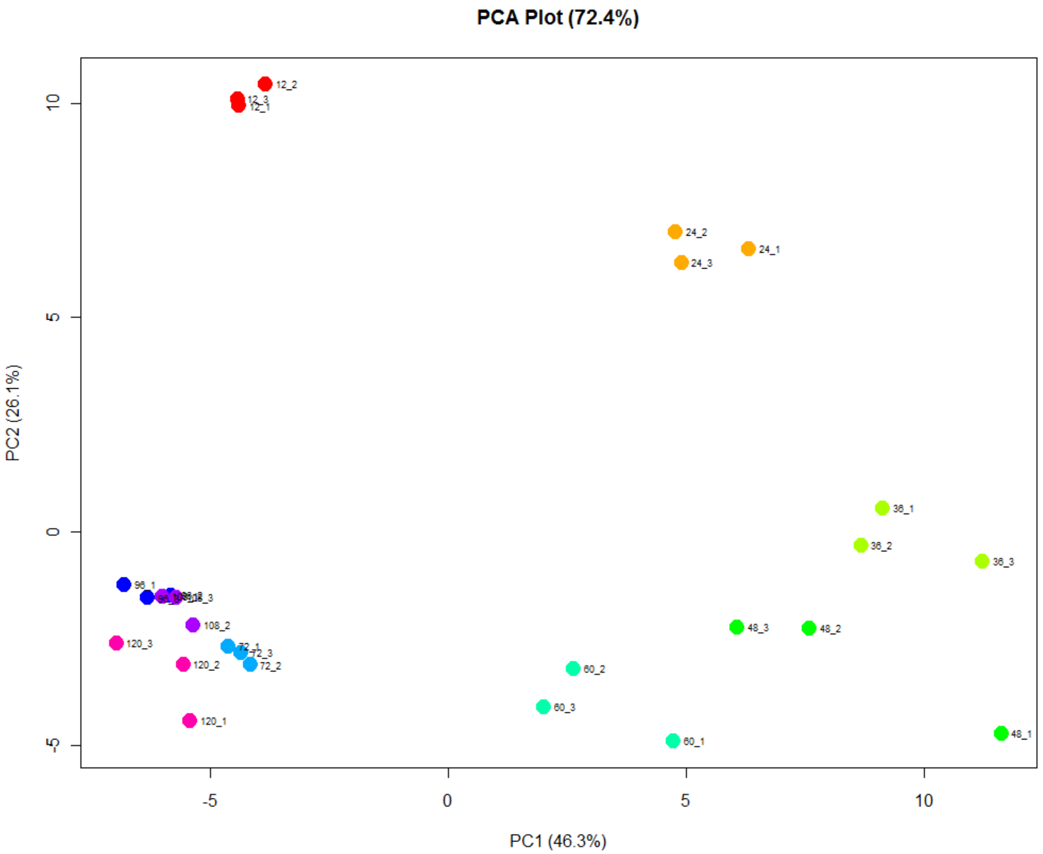

Supplemental figure 1C. Principal component analysis of extracellular metabolite datasets. Biological replicates are color coded and plotted along principal components 1 and 2 to visualize clustering and reproducibility.

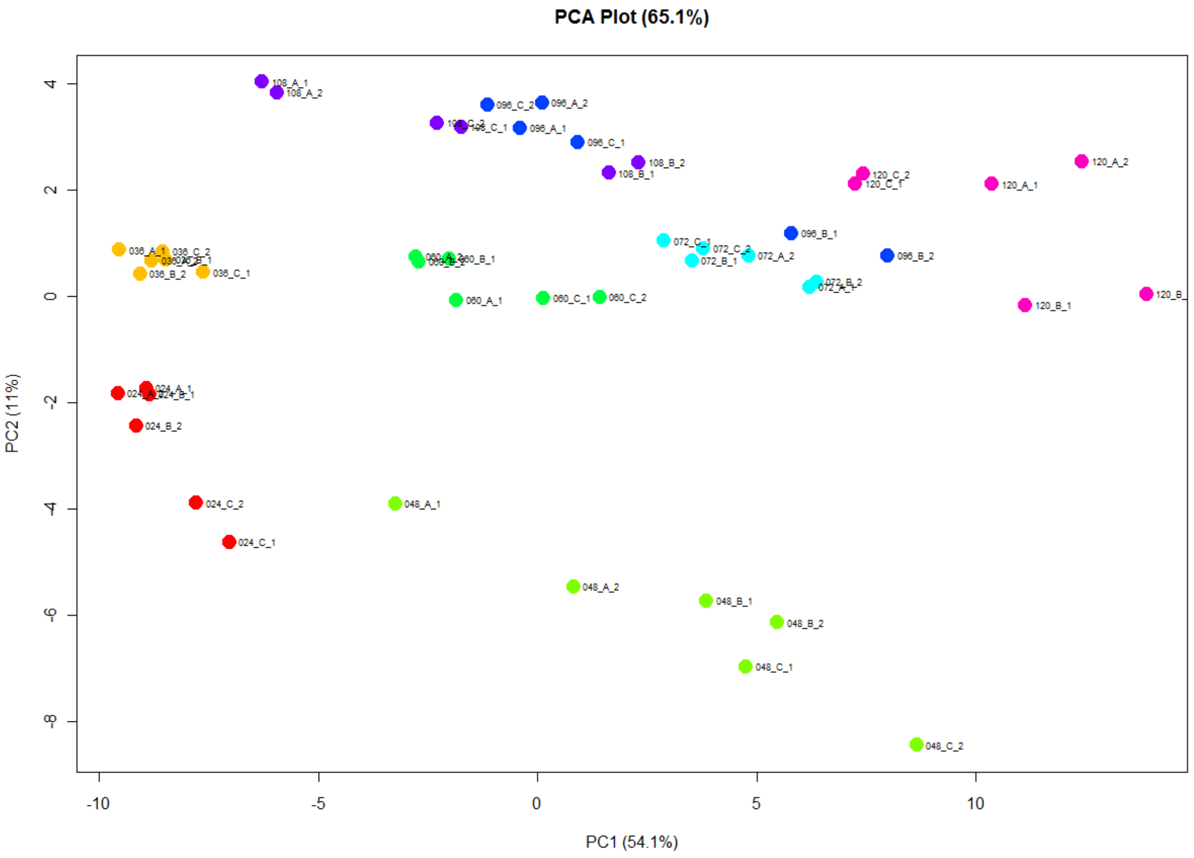

Supplemental figure 1D. Principal component analysis of intracellular polar metabolite datasets. Biological replicates are color coded and plotted along principal components 1 and 2 to visualize clustering and reproducibility.
