# Supplementary material for: Comprehensive Metabolomic, Lipidomic and Microscopic Profiling of Yarrowia lipolytica during Lipid Accumulation Identifies Targets for Increased Lipogenesis
Source: PLoS One. 2015 Apr 23;10(4):e0123188. doi: 10.1371/journal.pone.0123188 (PMC4408067; doi:10.1371/journal.pone.0123188)
Supplement: S1 Table — Substitution medium at T1 and T2 was 1% glutaraldehyde and 0.1% tannic acid in anhydrous acetone. Three washes in 100% acetone, and 1% osmium tetraoxide in 100% acetone followed as substitution media in T2–T3. (PDF) [file pone.0123188.s002.pdf]

Table S1. AFS procedure for *Y. lipolytica* cell suspensions.

Step	Temp (ºC)	Time (h)	Gradient (ºC h^{-1})
T1	-90	48	
S1		6	5
T2	-60	20	
S2		18	5
S2		12	5
T3	20	24	

Substitution medium at T1 and T2 was 1% glutaraldehyde and 0.1% tannic acid in anhydrous acetone. Three washes in 100% acetone, and 1% osmium tetraoxide in 100% acetone followed as substitution media in T2-T3.
