# Supplementary material for: Comprehensive Metabolomic, Lipidomic and Microscopic Profiling of Yarrowia lipolytica during Lipid Accumulation Identifies Targets for Increased Lipogenesis
Source: PLoS One. 2015 Apr 23;10(4):e0123188. doi: 10.1371/journal.pone.0123188 (PMC4408067; doi:10.1371/journal.pone.0123188)
Supplement: S2 Table — Measurements were obtained from z-stacks of cells stained for neutral lipids as in Fig 1. All volumes are in femtoliters. (PDF) [file pone.0123188.s003.pdf]

**Table S2. Volume of lipid droplets**

| Hour | **12** | **24** | **36** | **48** | **60** | **72** | **96** | **108** | **120** |
|---|---|---|---|---|---|---|---|---|---|
| Mean | 0.66 | 3.77 | 6.51 | 6.46 | 10.34 | 8.46 | 4.92 | 5.49 | 5.36 |
| Median | 0.09 | 2.93 | 5.86 | 4.68 | 9.37 | 7.11 | 1.80 | 2.43 | 3.01 |
| Standard deviation | 1.69 | 3.29 | 5.51 | 5.99 | 7.79 | 6.83 | 6.88 | 6.74 | 6.54 |

Measurements were obtained from z-stacks of cells stained for neutral lipids as in Figure 1. All volumes are in femtoliters.
